# Supplementary material for: Life Course Neighborhood Deprivation Effects on Body Mass Index: Quantifying the Importance of Selective Migration
Source: Int J Environ Res Public Health. 2021 Aug 6;18(16):8339. doi: 10.3390/ijerph18168339 (PMC8392830; doi:10.3390/ijerph18168339)
Supplement: Supplementary file 1 [file ijerph-18-08339-s001.zip › ijerph-1282528-SI.pdf]

Supplementary Table S1. Re-coding of residential mobility questions in the 1958 and 1970 cohorts into moved or not moved since last sweep variable.

	Sweep Interval	Survey question assessing of moved since last interval	Coding of moved variable
**1958 NCDS**			
16-23	23	Number of places lived since 16.	Moved: >=1<br>Not moved: <=1
23-33	33	No. addresses lived at since 16 + year moved into address (max 16).	Moved: >1 & year moved 1982-1991<br>Not moved: 0 or (1 & year moved 1956-1981)
33-42	42	Year moved into current address.	Moved: year 1992-2000<br>Not moved: year 1958-1991 or 9999
42-55	50*	Whether Cohort member living at same address as last interview/Jan2000.	Moved: Yes, same address<br>Not moved: No, different address
	55*	Whether living at same address as last interview.	Moved: Yes, same address OR Same address, incorrect details.<br>Not moved: No
**1970 BCS**			
16-26	26	Year moved into current address.	Moved: year 1987-1996<br>Not moved: year 1970-1986
26-34	30*	Lived somewhere else for 1mth+ since Ref data + year moved in.	Moved: yes + year 1997-2000<br>Not moved: year 1986-1996
	34*	Whether living at the same address during last interview.	Moved: yes<br>Not moved: No
34-42	42	Whether living at the same address during last interview.	Moved: Yes, same address<br>Not moved: No, different address

Supplementary Table S2: Direct effects modelled using structural equation modelling and multiple imputation (1970 cohort, n=18,639), Bonferroni corrections applied.

	Model 1. Area deprivation only		Model 2. Health selection only		Model 3. Both	
	Mean difference	*CI*	*Mean difference*	*CI*	*Mean difference*	*CI*
Fixed effects						
Body Mass Index (BMI) tracking:						
BMI16 → BMI26	0.70	0.66, 0.74	0.70	0.67, 0.73	0.70	0.65, 0.75
BMI26 → BMI34	0.41	0.38, 0.44	0.41	0.38, 0.44	0.41	0.37, 0.46
BMI34 → BMI42	0.49	0.46, 0.52	0.49	0.46, 0.52	0.49	0.45, 0.53
Townsend Area deprivation (TOWN) tracking:						
TOWN16 → TOWN26	0.93	0.91, 0.95	0.93	0.91, 0.95	0.93	0.91, 0.95
TOWN26 → TOWN34	0.98	0.94, 1.02	0.98	0.94, 1.02	0.96	0.91, 1.01
TOWN34 → TOWN42	0.66	0.62, 0.70	0.66	0.62, 0.70	0.66	0.61, 0.71
Area deprivation effect on BMI:						
TOWN16 → BMI26	0.04	-0.02, 0.07	-	-	0.04	-0.03, 0.11
TOWN26 → BMI34	0.06	0.00, 0.12	-	-	0.06	-0.01, 0.13
TOWN34 → BMI42	0.04	0.00, 0.08	-	-	0.04	-0.01, 0.09
Body Mass Index effect on Area deprivation:						
BMI16 → TOWN26	-	-	0.00	-0.04, 0.04	0.00	-0.05, 0.05
BMI26 → TOWN34	-	-	0.01	-0.02, 0.04	0.01	-0.03, 0.05
BMI34 → TOWN42	-	-	0.01	-0.01, 0.03	0.01	-0.01, 0.03
Model fit						
X^2 (df)	712.4 (17)		736.9 (17)		671.35 (14)	
RMSEA	0.047		0.048		0.050	
CFI	0.968		0.966		0.970	
TLI	0.950		0.958		0.942	
SRMR	0.042		0.047		0.041	

Supplementary Table S3: Direct effects modelled using structural equation modelling and multiple imputation (1958 cohort, n=18,555), Bonferroni corrections applied.

	Model 1.<br>Area deprivation only		Model 2.<br>Health selection only		Model 3.<br>Both	
	Mean difference	*CI*	*Mean difference*	*CI*	*Mean difference*	*CI*
Fixed effects						
Body Mass Index (BMI) tracking:						
BMI 16 → BMI 23	0.69	0.66, 0.72	0.69	0.66, 0.72	0.69	0.65, 0.73
BMI 23 → BMI 33	0.99	0.95, 1.03	0.99	0.95, 1.03	0.99	0.94, 1.04
BMI 33 → BMI 42	0.74	0.70, 0.78	0.74	0.70, 0.78	0.74	0.69, 0.79
BMI 42 → BMI 55	0.77	0.74, 0.80	0.77	0.74, 0.80	0.77	0.73, 0.81
Townsend Area deprivation (TOWN) tracking:						
TOWN16 → TOWN23	0.64	0.60, 0.68	0.64	0.60, 0.68	0.64	0.59, 0.69
TOWN23 → TOWN33	0.45	0.42, 0.48	0.45	0.42, 0.48	0.45	0.41, 0.49
TOWN33 → TOWN42	0.57	0.55, 0.59	0.57	0.55, 0.59	0.57	0.55, 0.59
TOWN42 → TOWN55	0.73	0.69, 0.77	0.73	0.70, 0.76	0.73	0.69, 0.77
Area deprivation effect on BMI:						
TOWN16 → BMI23	0.06	0.03, 0.09	-	-	0.06	0.02, 0.10
TOWN23 → BMI33	0.02	-0.03, 0.07	-	-	0.02	-0.03, 0.08
TOWN33 → BMI42	0.04	-0.02, 0.10	-	-	0.04	-0.03, 0.11
TOWN42 → BMI55	0.09	0.02, 0.16	-	-	0.09	0.01, 0.17
Body Mass Index effect on Area deprivation:						
BMI16 → TOWN23	-	-	0.01	-0.03, 0.05	0.01	-0.03, 0.05
BMI23 → TOWN33	-	-	0.05	0.02, 0.08	0.05	0.01, 0.09
BMI33 → TOWN42	-	-	0.01	-0.01, 0.03	0.01	-0.01, 0.03
BMI42 → TOWN55			0.01	0.00, 0.02	0.01	0.00, 0.02
Model fit						
X^2 (df)	2764.4 (31)		2752.6 (31)		2614.3 (27)	
RMSEA	0.069		0.069		0.072	
CFI	0.923		0.923		0.927	
TLI	0.890		0.891		0.881	
SRMR	0.074		0.074		0.070	

Supplementary Table S4. P-values for interaction tests of moving between intervals on area deprivation and health selection pathways.

	**Area deprivation effect** TOWN→BMI	**Health selection** BMI→TOWN
1970 British Cohort Study (BCS):		
Aged 16 to 26	0.278	0.813
Aged 26 to 34	0.321	0.685
Aged 34 to 42	0.569	0.272
National Child Development Study (NCDS):		
Aged 16 to 26	0.285	0.482
Aged 26 to 34	0.828	0.407
Aged 34 to 42	0.559	0.956
Aged 42 to 55	0.690	0.199
